# P-2028. Utilizing analytics dashboards to improve blood culture contamination rates

**DOI:** 10.1093/ofid/ofaf695.2192

**Published:** 2026-01-11

**Authors:** Jonathan Berback, Elisa Moyer

**Affiliations:** Jefferson Lehigh Valley, Allentown, PA; LVHN part of Jefferson Health, Allentown, Pennsylvania

## Abstract

**Background:**

Blood culture contamination rates, like many other indicators, suffered over the course of the COVID-19 Pandemic. In 2022, a renewed focus was placed on reducing contamination rates. A diversion device was deployed, and contamination rates were tracked with passive lab-based criteria. Each contamination event required chart abstraction to gather details and provide feedback.

**Figure:**
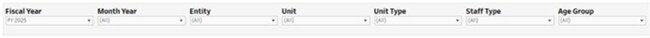
Dashboard Filters End users can use these fields to get the data most applicable to them.

**Figure:**
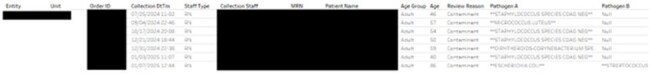
Data Points For users with access to PHI, all of these details are made available.

**Methods:**

A process was created in the EHR which allowed Infection Preventionists to classify blood culture contamination events. By completing this process within the EHR all the key details associated with each blood culture collection were captured. A report was also created to generate the total denominator of all blood cultures collected. This data was then mapped to an analytics dashboard allowing us to share real time contamination rates. The dashboard allows drilling down to the hospital, department, and staff member levels, identifying the organism, distinguishing between pediatric and adult patients, and differentiating between phlebotomy and nursing data.

**Figure:**
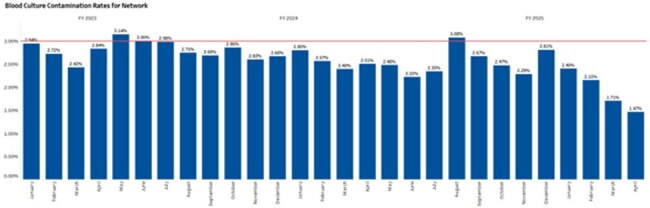
Contamination Rate Graph The graph provides a quick visual for identifying trends in the data.

**Results:**

Since the rollout, our dashboard has been viewed ∼1400 times by senior leadership, quality representatives, unit leaders and educators. Awareness and utilization of the dashboard has improved since its roll out and so too has the overall contamination rate.

Pre dashboard (January 2023- April 2024) contamination totals 2,121 contamination out of 77,812 Cultures = 2.73% average contamination rate.

Post Dashboard (May 2024 – April 2025) contamination totals 1,369 contaminations out of 58,434 Cultures = 2.34% average contamination rate.

This reduction is also statistically significant. A two-proportion Z-test was used to determine that it’s very unlikely to be due to random chance (Z-value=4.51). For a two tailed test, the p-value is then calculated as 0.000006 or p < 0.00001.

**Conclusion:**

With continued growth of our network, actionable data is critical to assist with quality care delivery. Front line staff want to be active participants in these types of metric based goals. Creating a self-service platform has proven to be a staff satisfier as it allows for more individuals to champion the issue of blood culture contamination. Colleague engagement has been key to the early success of our intervention.

**Disclosures:**

All Authors: No reported disclosures

